# *Pyropia yezoensis* Protein Supplementation Prevents Dexamethasone-Induced Muscle Atrophy in C57BL/6 Mice

**DOI:** 10.3390/md16090328

**Published:** 2018-09-11

**Authors:** Min-Kyeong Lee, Jeong-Wook Choi, Youn Hee Choi, Taek-Jeong Nam

**Affiliations:** 1 Institute of Fisheries Sciences, Pukyong National University, Busan 46041, Korea; 3633234@hanmail.net (M.-K.L.); wook8309@naver.com (J.-W.C.); 2 Department of Marine Bio-Materials & Aquaculture, Pukyong National University, Busan 48513, Korea; 3 Department of Food Science and Nutrition, Pukyong National University, Busan 48513, Korea

**Keywords:** dexamethasone, muscle atrophy, protein breakdown, protein synthesis, *Pyropia yezoensis*

## Abstract

We investigated the protective effects of *Pyropia yezoensis* crude protein (PYCP) against dexamethasone (DEX)-induced myotube atrophy and its underlying mechanisms. DEX (3 mg/kg body weight, intraperitoneal injection) and PYCP (150 and 300 mg/kg body weight, oral) were administrated to mice for 18 days, and the effects of PYCP on DEX-induced muscle atrophy were evaluated. Body weight, calf thickness, and gastrocnemius and tibialis anterior muscle weight were significantly decreased by DEX administration (*p* < 0.05), while PYCP supplementation effectively prevented the DEX-induced decrease in body weight, calf thickness, and muscle weight. PYCP supplementation also attenuated the DEX-induced increase in serum glucose, creatine kinase, and lactate dehydrogenase levels. Additionally, PYCP supplementation reversed DEX-induced muscle atrophy via the regulation of the insulin-like growth factor-I/protein kinase B/rapamycin-sensitive mTOR complex I/forkhead box O signaling pathway. The mechanistic investigation revealed that PYCP inhibited the ubiquitin-proteasome and autophagy-lysosome pathways in DEX-administrated C57BL/6 mice. These findings demonstrated that PYCP increased protein synthesis and decreased protein breakdown to prevent muscle atrophy. Therefore, PYCP supplementation appears to be useful for preventing muscle atrophy.

## 1. Introduction

Skeletal muscle atrophy occurs in a variety of conditions, such as muscle inactivity (e.g., joint immobilization and prolonged bed rest), fasting, multiple disease states, age-related atrophy, and glucocorticoid (GC) treatment [1,2]. GC is commonly used in the treatment of various diseases, such as bronchial asthma, rheumatoid arthritis, and systemic lupus erythematosus [3,4,5]. GC effectively inhibits inflammation in various inflammatory diseases; however, high doses and sustained administration leads to various progressive side effects [6,7], among which, muscle atrophy is a particularly serious complication with profound effects on daily life, such as physical activity, due to weakness of proximal skeletal muscle [8]. The mechanisms associated with DEX-induced muscle atrophy are not fully understood, but some studies suggest that inhibition of protein anabolism or promotion of protein catabolism may be responsible [9,10].

Several studies have demonstrated that the insulin-like growth factor-I (IGF-I)-mediated phosphoinositide 3-kinase/protein kinase B/rapamycin-sensitive mTOR complex I (PI3K/Akt/mTORC1) signaling pathway has an important role in increasing protein synthesis associated with muscle hypertrophy [11,12]. IGF-I is a circulating growth factor produced locally in many tissues, including skeletal muscle [13]. IGF-I increases protein synthesis by activating downstream targets of the PI3K/Akt/mTORC1 signaling pathway [14]. Studies have demonstrated that GC promotes catabolism by inhibiting the PI3K/Akt/mTORC1 pathway, mediating IGF-I assimilation. For example, GC has been shown to reduce IRS-1 protein levels, the first upstream factor in the PI3K/Akt cascade, resulting from the stimulation of C1-Ten, a protein tyrosine phosphatase that promotes IRS-1 degradation [15,16]. In addition, GC can block the PI3K/Akt pathway by inducing miR1 expression [17]. Recent studies have shown that the inhibition of mTORC1 complex by GC is induced by the transcription of the mTORC1 repressors regulated in development and DNA damage response 1 (REDD1) and Kruppel-like factor 15 (KLF-15) [18]. These findings suggest that GC-induced skeletal muscle atrophy could be mediated by a decrease in IGF-I/Akt/mTORC1 activity in skeletal muscle.

The interaction between protein synthesis and degradation is regulated not only by the Akt/mTOR pathway, but also by the Akt/forkhead box O (FoxO pathway) [19]. FoxO, a transcription factor in skeletal muscle, has three isoforms: FoxO1, FoxO3, and FoxO4. Studies have shown that FoxO activation upregulates eukaryotic initiation factor 4E-binding protein 1 (4E-BP1) and downregulates mTORC1 [20]. Thus, protein synthesis is further suppressed by the inhibition of Akt and activation of FoxO when activating protein degradation. Akt phosphorylates FoxO to increase FoxO migration from the nucleus to the cytoplasm [21]. In a study of muscle atrophy, Akt inhibition showed a marked increase in nuclear translocation of FoxO [22]. There is some evidence that the transcription factor FoxO has an important role in the effect of GC on the catabolic activity of skeletal muscle. First, skeletal muscle exposed to GC shows increased expression of FoxO transcription factors, especially FoxO1 and FoxO3a [23,24,25]. Second, overexpression of FoxO in in vivo and in vitro models leads to the activation of several atrogenes, such as atrogin-1/Muscle Atrophy F-Box (MAFbx), muscle RING finger 1 (MuRF1), and cathepsin-L, causing atrophy [26,27]. Finally, overexpression of dominant negative forms of FoxO3a prevents muscle atrophy by inhibiting the expression of GC-induced atrogin-1/MAFbx [27]. Thus, degradation of muscle proteins by GC stimulation is ultimately triggered by the activation of cellular proteolytic systems, such as the ubiquitin-proteasome and autophagy-lysosome systems [28]. 

*Pyropia yezoensis* Ueda (*P. yezoensis*; Bangiaceae, Rhodophyta) is an economically important marine red alga cultivated primarily in Asian countries (e.g., Korea, China, and Japan) [29]. *Pyropia yezoensis* contains 25–50% protein, 20–40% carbohydrate, and various vitamins and essential minerals on a dry matter basis [30]. Further, it shows medicinal benefits, including anti-photoaging, anti-inflammatory, and antioxidant effects. A recent study demonstrated that cyclophilin-type peptidylprolyl *cis-trans* isomerase from *P. yezoensis* helped prevent hydrogen peroxide-induced oxidative stress in HepG2 cells [31]. In addition, *P. yezoensis* glycoprotein was found to prevent D-galactosamin/lipopolysaccharide-induced hepatotoxicity in Hepa1c1c7 cells and a rat model [32,33]. However, the efficacy of *P. yezoensis* protein supplementation in the prevention of DEX-induced muscle atrophy remains unclear. 

Therefore, we investigated whether the *P. yezoensis* protein could prevent skeletal muscle atrophy induced by DEX exposure in C57BL/6 mice. In addition, candidate mechanisms underlying the inhibition of skeletal muscle atrophy by *Pyropia yezoensis* crude protein (PYCP) supplementation were considered.

## 2. Results

### 2.1. PYCP Supplementation Attenuates DEX-Induced Loss of Body Weight

To evaluate the efficacy of DEX administration (3 mg/kg body weight) to induce muscle atrophy, the body weight of the mice was recorded every day for 18 days. There was no difference in body weight among the groups before DEX treatment. During the DEX treatment period, dietary intake of PYCP in the DEX group did not differ from that in the control group; however, the DEX group had a significantly lower body weight than the control group (*p* < 0.05). Moreover, PYCP supplementation effectively prevented the DEX-induced decrease in body weight (Table 1).

### 2.2. PYCP Supplementation Attenuates DEX-Induced Loss of Calf Thickness and Muscle Weight

**Figure 1:**
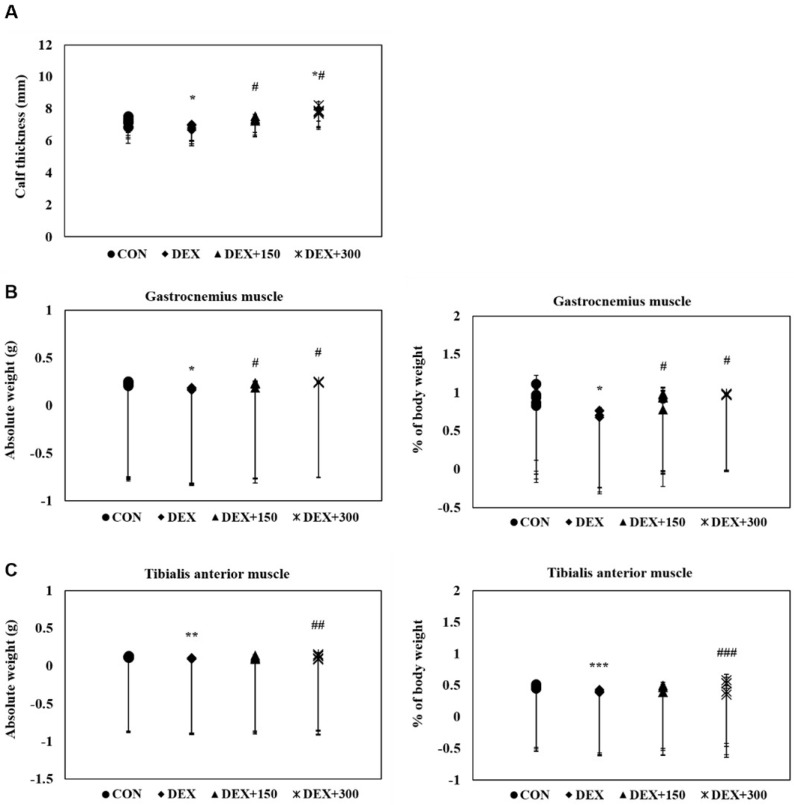

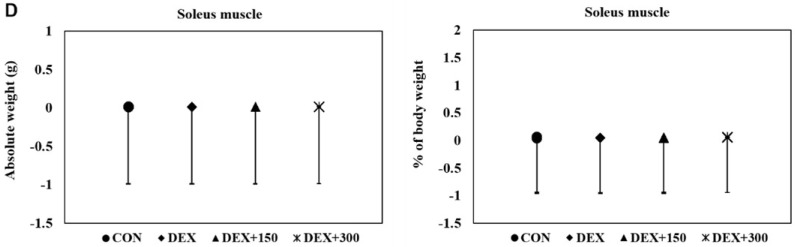
Effect of PYCP supplementation on changes in calf thickness and muscle weight in DEX-stimulated C57BL/6 mice. (**A**) The calf thickness of the left hind limb was measured after exposing the muscle at sacrifice, as described in the materials and methods. (**B**–**D**) The gastrocnemius, soleus, and tibialis anterior muscles were carefully separated from the tibia and fibula bones and weighed (absolute wet weight). The results are presented as the means ± standard deviation of five mice. * *p* < 0.05, ** *p* < 0.01, *** *p* < 0.001 vs. the corresponding control group; ^#^ *p* < 0.05, ^##^ *p* < 0.01, ^###^ *p* < 0.001 vs. the corresponding DEX-only treatment.

To determine the protective effects of PYCP on DEX-stimulated muscle loss in C57BL/6 mice, calf thickness and muscle weight were measured immediately after biopsy. C57BL/6 mice stimulated with DEX alone exhibited a marked decrease in calf thickness compared with untreated control mice (Figure 1A). However, PYCP supplementation effectively prevented the DEX-induced decrease in calf thickness. Additionally, C57BL/6 mice stimulated with DEX alone exhibited a marked decrease in gastrocnemius and tibialis anterior muscle weight compared with untreated control mice, which was attenuated by PYCP supplementation (Figure 1B,C). No significant changes in soleus muscle weight were observed (Figure 1D).

### 2.3. Changes in Serum Biochemistry in Mice with DEX-Induced Muscle Atrophy 

**Figure 2:**
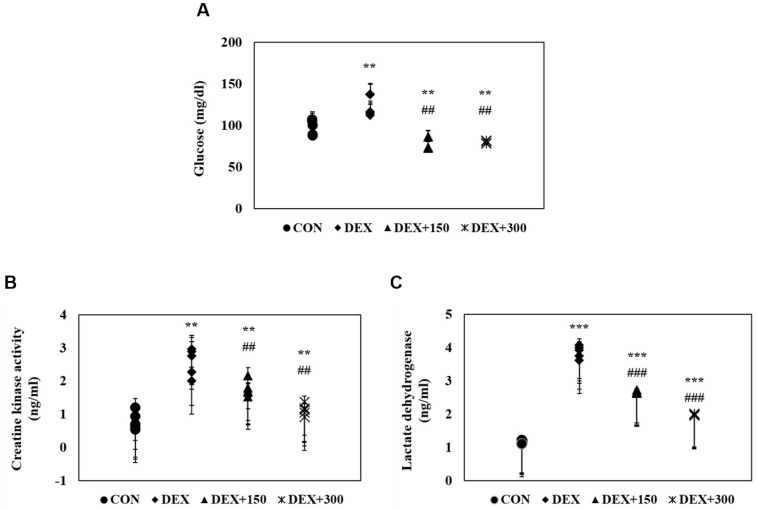
Effects of PYCP supplementation on serum biochemistry, including (**A**) glucose, (**B**) creatine kinase, and (**C**) lactate dehydrogenase levels, assessed by ELISA. Data represent mean ± standard deviation of two independent experiments (n = 5/group). ** *p* < 0.01, *** *p* < 0.001 vs. the corresponding control group; ^##^ *p* < 0.01, ^###^ *p* < 0.001 vs. the corresponding DEX-only treatment group.

To determine the glucose homeostasis of DEX-treated C57BL/6 mice, serum glucose levels were measured by enzyme-linked immunosorbent assay (ELISA). C57BL/6 mice treated with DEX alone exhibited a marked increase in serum glucose levels compared with untreated control mice (Figure 2A). However, DEX-induced upregulation of serum glucose levels was decreased by PYCP supplementation. To determine the creatine kinase (CK) and lactate dehydrogenase (LDH) activities of DEX-treated C57BL/6 mice, serum CK and LDH levels were measured by ELISA. CK and LDH levels were markedly increased in DEX-treated mice, indicating that muscle atrophy was induced by DEX treatment (Figure 2B,C). However, supplementation of PYCP markedly reduced CK and LDH levels in DEX-treated mice. These results indicate that PYCP has a protective effect on muscle atrophy.

### 2.4. PYCP Supplementation Upregulates the Decrease in DEX-Induced IGF-IR and IRS-1 Phosphorylation in Gastrocnemius Muscle

**Figure 3:**
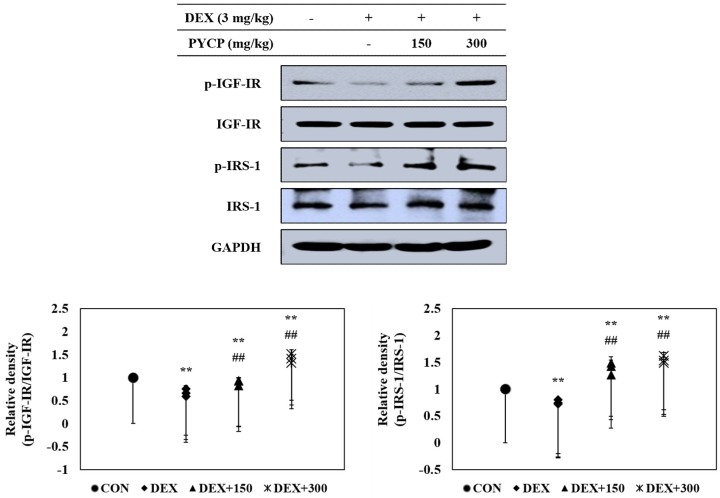
Effects of PYCP supplementation on IGF-IR and IRS-1 phosphorylation in gastrocnemius muscle. The protein levels of p-IGF-IR, IGF-IR, p-IRS-1, and IRS-1 were assessed by Western blot analysis. GAPDH was used as an internal standard. Data represent mean ± standard deviation of three independent experiments (n = 5/group). ** *p* < 0.01 vs. the corresponding control group; ^##^ *p* < 0.01 vs. the corresponding DEX-only treatment group.

The effects of PYCP on the IGF-I pathway in DEX-administrated C57BL/6 mice were determined by Western blot analysis. DEX administration significantly decreased the IGF-IR and IRS-1 phosphorylation in gastrocnemius muscle (*p* < 0.05) (Figure 3), which was attenuated by PYCP supplementation in a dose-dependent manner.

### 2.5. PYCP Supplementation Upregulates the Decrease in the DEX-Induced Akt/mTORC1 Signaling Pathway in Gastrocnemius Muscle

**Figure 4:**
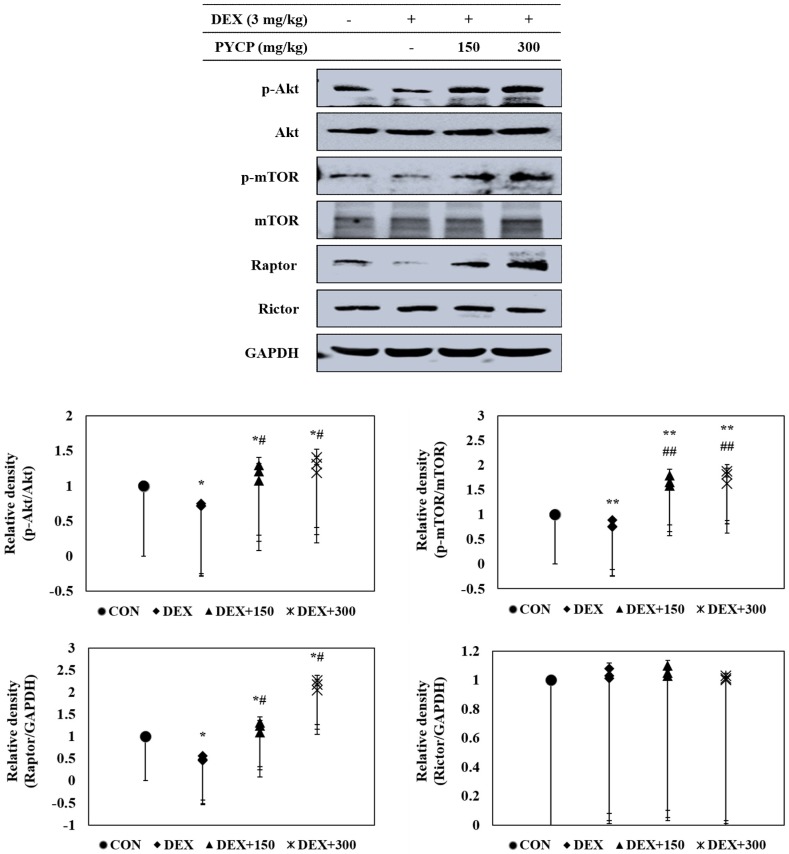
Effects of PYCP supplementation on the Akt/mTOR signaling pathway in gastrocnemius muscle. The protein levels of p-Akt, Akt, p-mTOR, mTOR, Raptor, and Rictor were assessed by Western blot analysis. GAPDH was used as an internal standard. Data represent mean ± standard deviation of three independent experiments (n = 5/group). * *p* < 0.05, ** *p* < 0.01 vs. the corresponding control group; ^#^ *p* < 0.05, ^##^ *p* < 0.01 vs. the corresponding DEX-only treatment group.

To further clarify the molecular mechanisms of the regulatory effects of PYCP on IGF-I activation in DEX-stimulated C57BL/6 mice, the effect of PYCP on the phosphorylation of the Akt/mTOR signaling pathway was examined by Western blot analysis. DEX administration decreased Akt and mTOR phosphorylation in gastrocnemius muscle (Figure 4), which was attenuated by PYCP supplementation in a dose-dependent manner. To determine whether PYCP induced changes in mTORC1 and mTORC2 protein levels in DEX-stimulated C57BL/6 mice, Raptor and Rictor protein levels were examined. As expected, DEX administration significantly decreased Raptor expression compared with the control group (*p* < 0.05), which was attenuated by PYCP supplementation. By contrast, no significant differences in Rictor levels were observed compared with the control group. These results reveal that PYCP supplementation suppresses DEX-induced muscle atrophy via mTORC1 signaling activation.

### 2.6. PYCP Supplementation Upregulates the Decrease in DEX-Induced p70S6K and 4E-BP1 Phosphorylation in Gastrocnemius Muscle

**Figure 5:**
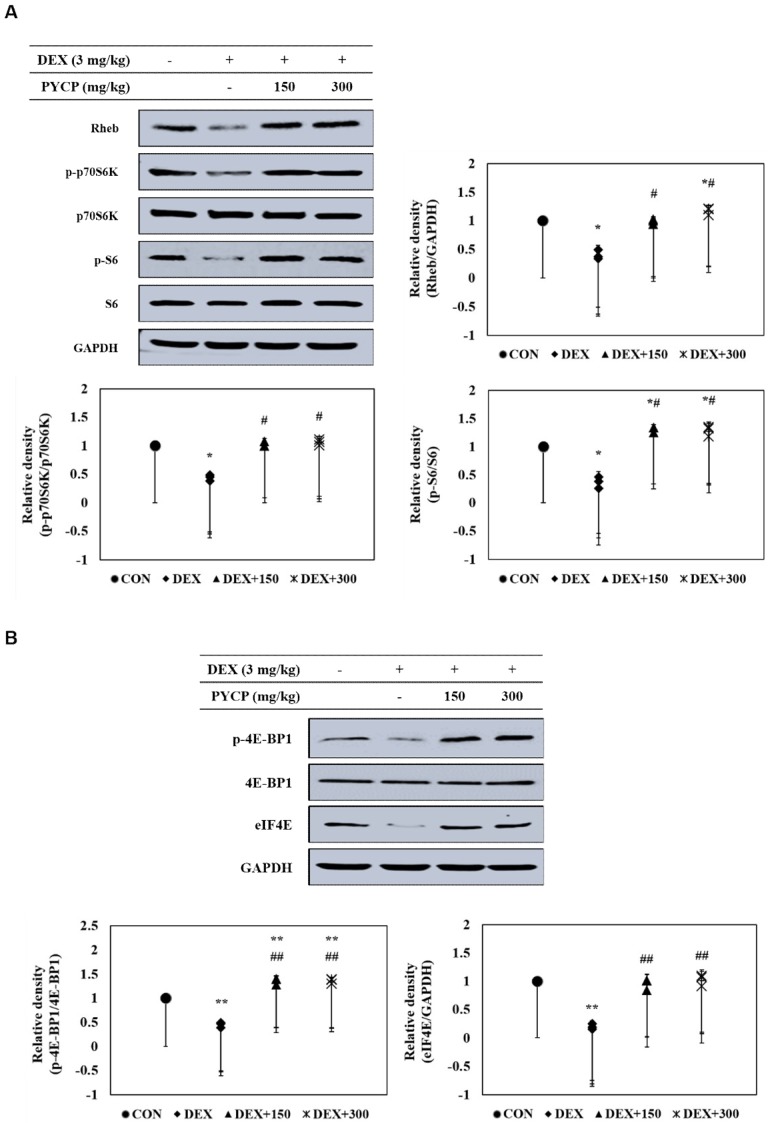
Effects of PYCP on the mTORC1 downstream signaling components in gastrocnemius muscle. (**A**) Rheb, p-p70S6K, p70S6K, p-S6, and S6 protein levels were assessed by Western blot analysis. (**B**) p-4E-BP1, 4E-BP1, and eIF4E protein levels were assessed by Western blot analysis. GAPDH was used as an internal standard. Data represent mean ± standard deviation of three independent experiments (n = 5/group). * *p* < 0.05, ** *p* < 0.01 vs. the corresponding control group; ^#^ *p* < 0.05, ^##^ *p* < 0.01 vs. the corresponding DEX-only treatment group.

To further clarify the downstream signals regulated by mTORC1 signaling activation, the phosphorylation levels of p70S6K and 4E-BP1 pathway members were examined in C57BL/6 mice. DEX administration significantly decreased Rheb protein levels compared with the untreated control group (*p* < 0.05) (Figure 5A), which was attenuated by PYCP supplementation in a dose-dependent manner. In addition, p70S6K, S6, and 4E-BP1 phosphorylation and eIF4E protein expression were significantly decreased by DEX administration, but attenuated by PYCP supplementation (*p* < 0.05, *p* < 0.01) (Figure 5A,B).

### 2.7. PYCP Supplementation Downregulates the Increase in DEX-Induced FoxO1 and FoxO3a Activation in Gastrocnemius Muscle

**Figure 6:**
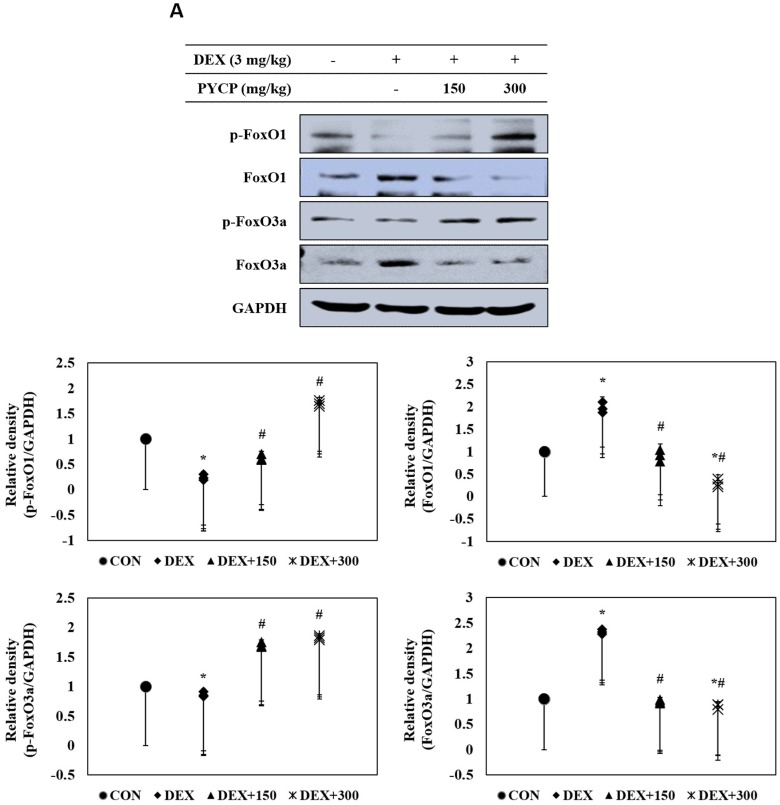

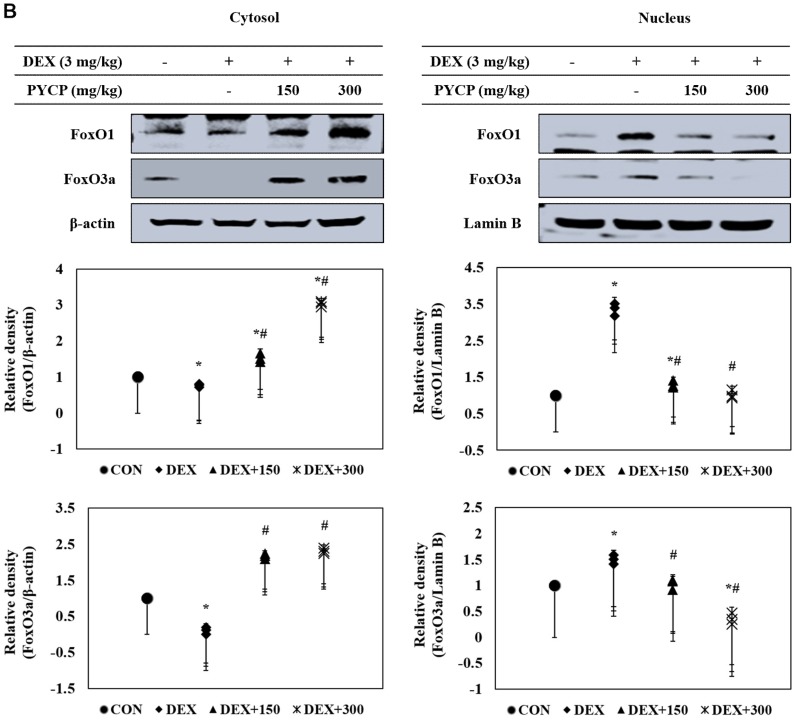
Effects of PYCP supplementation on the activation and translocation of FoxO1 and FoxO3a in gastrocnemius muscle. (**A**) p-FoxO1, FoxO1, p-FoxO3a, and FoxO3a protein levels were examined by Western blot analysis. GAPDH was used as an internal standard; (**B**) FoxO1 and FoxO3a levels were measured in cellular cytosolic and nuclear fractions by Western blot analysis. β-actin, and lamin B were used as internal standards. Data represent mean ± standard deviation of three independent experiments (n = 5/group). * *p* < 0.05 vs. the corresponding control group; ^#^ *p* < 0.05 vs. the corresponding DEX-only treatment group.

To investigate the mechanistic effects of the anti-muscle atrophy activity of PYCP on transcriptional regulation, we measured FoxO activation by Western blot analysis. DEX administration markedly increased total FoxO1 and FoxO3a expression in gastrocnemius muscle (Figure 6A), which was prevented by PYCP supplementation in a dose-dependent manner. In addition, DEX treatment resulted in decreased FoxO1 and FoxO3a phosphorylation, which was attenuated by PYCP supplementation in a dose-dependent manner (Figure 6A). FoxO dephosphorylation causes its translocation from the cytoplasm to the nucleus. DEX administration significantly increased the levels of nuclear FoxO1 and FoxO3a (*p* < 0.05) (Figure 6B), which was attenuated by PYCP supplementation in a dose-dependent manner.

### 2.8. PYCP Supplementation Inhibits the DEX-Induced Activation of the Ubiquitin-Proteasome System in Gastrocnemius Muscle

**Figure 7:**
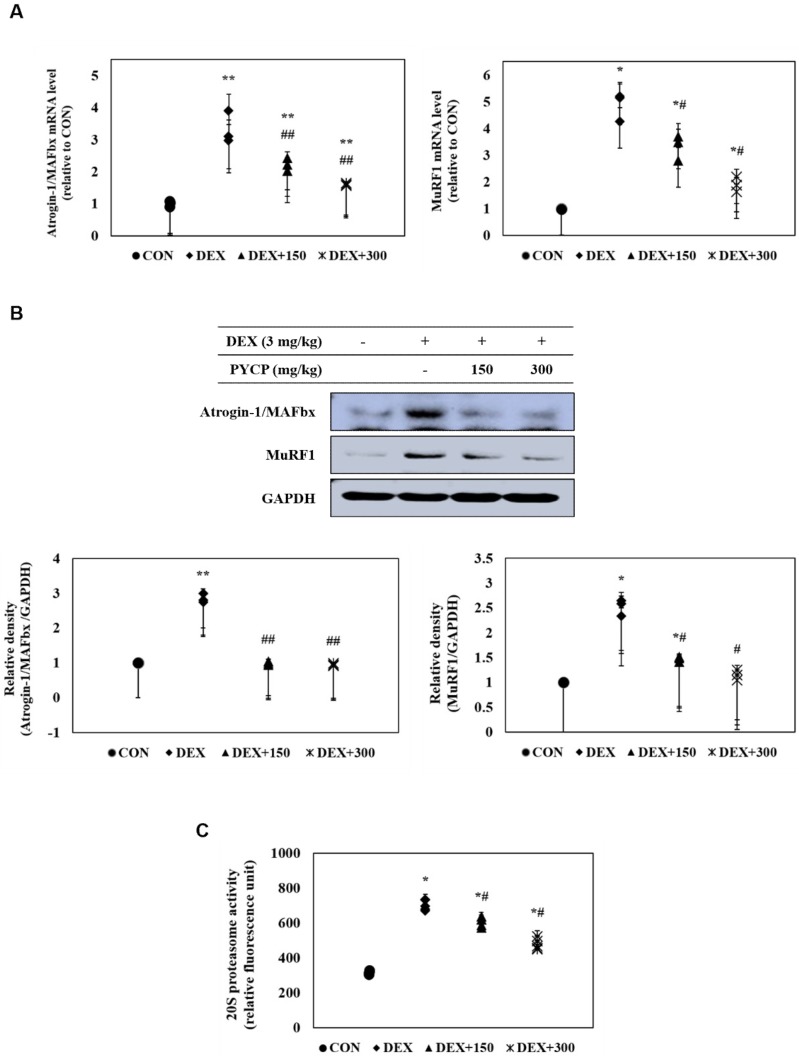
Effects of PYCP supplementation on the ubiquitin-proteasome system in gastrocnemius muscle. (**A**,**B**) Atrogin-1/MAFbx and MuRF1 mRNA and protein levels were assessed by real-time PCR and Western blot analysis. GAPDH was used as an internal standard; (**C**) 20S proteasomal activity was assessed by the detection of AMC in gastrocnemius muscle post cleavage from the peptide LLVY-AMC. Data represent mean ± standard deviation of three independent experiments (n = 5/group). * *p* < 0.05, ** *p* < 0.01 vs. the corresponding control group; ^#^ *p* < 0.05, ^##^ *p* < 0.01 vs. the corresponding DEX-only treatment group.

The effects of PYCP on atrogin-1/MAFbx and MuRF1 expression in DEX-treated C57BL/6 mice were assessed with real-time PCR and Western blot analysis. DEX administration markedly increased atrogin-1/MAFbx and MuRF1 mRNA and protein expression in gastrocnemius muscle (Figure 7A,B). However, supplementation of PYCP inhibited DEX-induced increases of atrogin-1/MAFbx and MuRF1 in a dose-dependent manner. In addition, to examine the protective effect of PYCP on 20S proteasome activity, a 7-amino-4-methylcounmarin (AMC) fluorescent substance was used. DEX administration significantly increased 20S proteasome activity, which was attenuated by PYCP supplementation in a dose-dependent manner (*p* < 0.05) (Figure 7C).

### 2.9. PYCP Supplementation Inhibits the Activation of the DEX-Induced Autophagy-Lysosome System in Gastrocnemius Muscle

**Figure 8:**
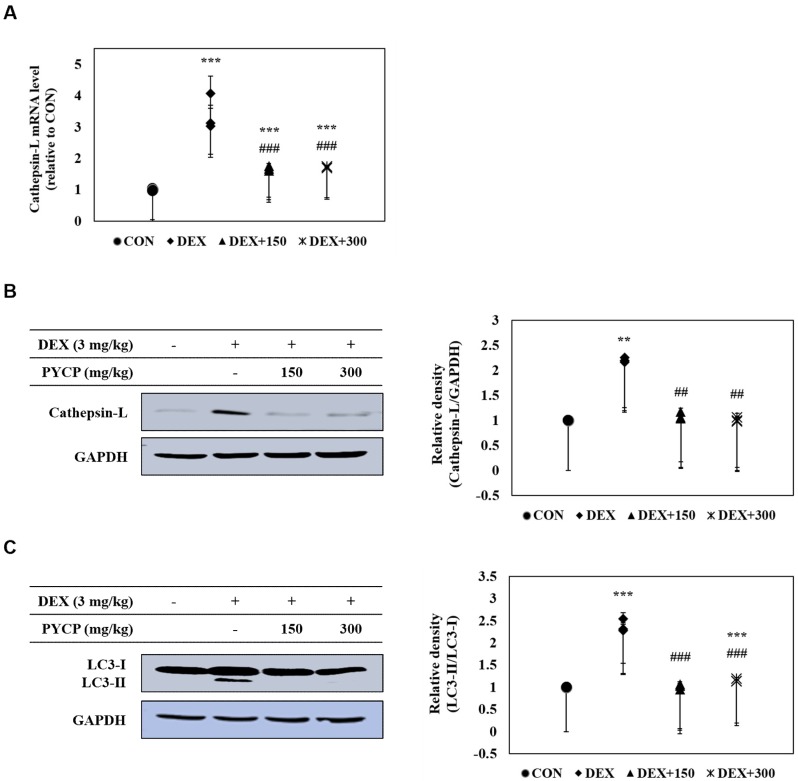

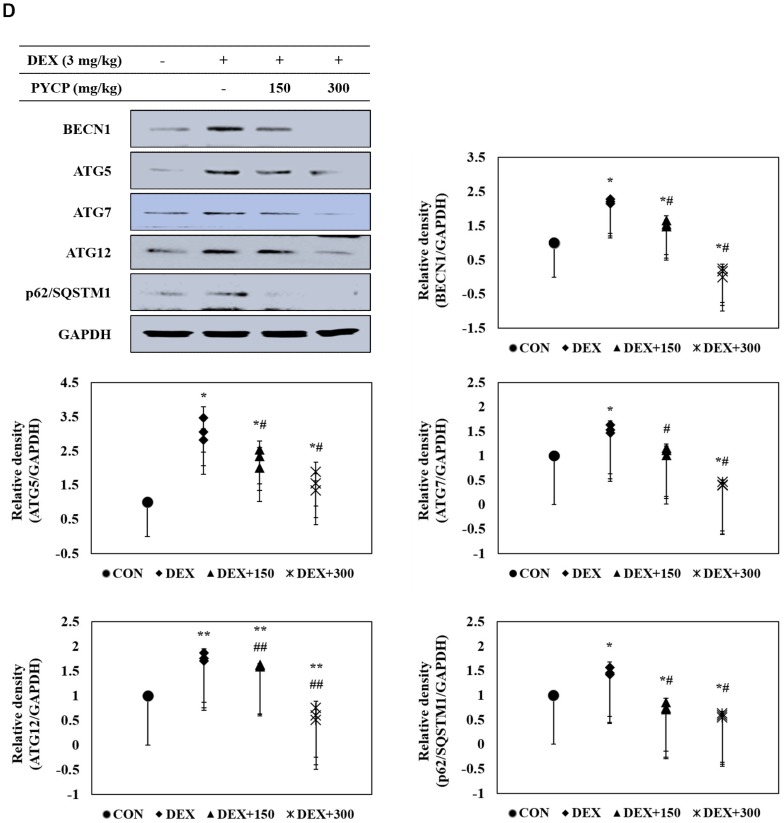
Effects of PYCP supplementation on the autophagy-lysosome system in gastrocnemius muscle. (**A**,**B**) Cathepsin-L mRNA and protein levels were assessed with real-time PCR and Western blot analysis; (**C**,**D**) protein levels of autophagy-related genes (i.e., LC3-I/LC3-II, BECN1, ATG5, ATG7, ATG12, and p62/SQSTM1) assessed by Western blot analysis. GAPDH was used as an internal standard. Data represent mean ± standard deviation of three independent experiments (n = 5/group). * *p* < 0.05, ** *p* < 0.01, *** *p* < 0.001 vs. the corresponding control group; ^#^ *p* < 0.05, ^##^ *p* < 0.01, ^###^ *p* < 0.001 vs. the corresponding DEX-only treatment group.

The effects of PYCP on the expression of cathepsin-L and autophagy-related genes in DEX-administrated C57BL/6 mice were determined with real-time PCR and Western blot analysis. DEX administration significantly increased cathepsin-L mRNA and protein expression in gastrocnemius muscle tissue (*p* < 0.01, *p* < 0.001) (Figure 8A,B), which was inhibited by PYCP supplementation in a dose-dependent manner. In addition, the protein levels of autophagy-related genes (i.e., LC3-I/II, BECN1, ATG5, ATG7, ATG12, and p62/SQSTM1) were increased by DEX administration, which was attenuated by PYCP supplementation (Figure 8C,D).

## 3. Discussion

Recent studies have demonstrated that the regulation of IGF-I and its downstream pathway, Akt/mTORC1, has a pivotal role in the prevention of muscle atrophy by inhibiting the proteolytic system [34]. Therefore, we investigated the protective role of PYCP against DEX-induced muscle atrophy in C57BL/6 mice and the mechanisms underlying the anti-atrophy effects of PYCP in mice administered DEX.

DEX-induced muscle atrophy is characterized by decreased muscle size and protein content, loss of cellular organelles, reduced muscle strength, and fatigue resistance [24,35]. In this study, oral supplementation of PYCP increased body weight, calf thickness, and muscle weight in C57BL/6 mice administered DEX. In particular, PYCP supplementation prevented the DEX-induced reductions in gastrocnemius and tibialis anterior muscle weight; however, no significant changes in soleus muscle weight were observed. These results correspond with those of an earlier study that reported the increased susceptibility of glycolytic muscles (i.e., tibialis anterior) to GC-induced muscle atrophy compared to oxidative muscles (i.e., soleus) [36]. 

The enzymes CK and LDH are released during tissue damage, and are serum markers of common injury and disease, particularly muscle damage [37,38]. In this study, marked increases in serum CK and LDH levels were observed along with DEX-related catabolic muscle atrophic changes, as previously demonstrated; however, PYCP treatment markedly inhibited these increases in a dose-dependent manner, demonstrating that PYCP can prevent muscle atrophy caused by DEX exposure.

DEX-induced muscle atrophy is promoted by the activation of proteolytic systems (e.g., ubiquitin-proteasome pathway, autophagy-lysosome pathway, and calcium-dependent pathway) as a result of increased protein degradation and decreased protein synthesis [39]. Stimulation of the proteolytic system by DEX is induced by increased expression of several atrogenes, such as atrogin-1/MAFbx, MuRF1, and cathepsin-L, which are regulated by the FoxO transcription factor. In addition, the inhibitory effect of DEX on muscle protein synthesis is mainly related to inhibition of the IGF-I/Akt/mTORC1 signaling pathway [40,41,42]. A recent study demonstrated an unclear effect of *P. yezoensis* protein on muscle function; however, our previous study demonstrated that *P. yezoensis* protein regulated the IGF-I pathway [43,44]. Thus, in this study, we hypothesized that PYCP would prevent muscle atrophy by regulating the IGF-I signaling pathway in DEX-induced muscle atrophy.

DEX induces muscle atrophy by inhibiting the production of IGF-I, a growth factor that stimulates muscle growth by reducing proteolysis and increasing protein synthesis [45,46]. Therefore, we believe that boosting the IGF-I-dependent response to counteract skeletal muscle atrophy is a promising strategy for preventing DEX-induced muscle atrophy. Studies have shown that the action of IGF-I toward muscle growth is mediated through IGF-IR [14]. The present study revealed markedly decreased IGF-IR and IRS-1 phosphorylation after DEX exposure, which was prevented by PYCP supplementation. These results suggest that the regulation of DEX-induced muscle atrophy by PYCP could be associated with the IGF-I pathway. IGF-I has an important role in reducing muscle atrophy by stimulating muscle protein synthesis and hypertrophy via Akt/mTOR pathway activation [47]. The results revealed decreased Akt and mTOR phosphorylation in gastrocnemius muscle after DEX exposure, which was upregulated by PYCP treatment. mTOR is a serine-threonine kinase that forms two distinct complexes, mTORC1 and mTORC2 [21]. DEX-induced muscle atrophy is mainly triggered by inhibition of mTORC1, a kinase involved in 4E-BP1 and S6K phosphorylation [48]. Recent studies have demonstrated that the reduction of mTOR phosphorylation by DEX is mainly caused by mTORC1 inhibition [49], which is the result of REDD1 and KLF-15 transcription, two repressors of the mTORC1 signaling pathway [50]. The present study demonstrated that PYCP activated Raptor expression in DEX-stimulated C57BL/6 mice. These results suggest that PYCP inhibits muscle atrophy through the activation of the mTORC1 signaling pathway in DEX-stimulated C57BL/6 mice. The activation of the Akt/mTORC1 pathway by IGF-I stimulates protein translation and synthesis via p70S6K activation and 4E-BP1 inhibition [22]. DEX inhibits the stimulatory action of IGF-I on 4E-BP1 and S6K phosphorylation, which has an important role in protein synthesis machinery by controlling the initiation phase of mRNA translation [51,52]. This study demonstrated that p70S6K, S6, and 4E-BP1 phosphorylation and eIF4E expression, which were all reduced by DEX administration, were increased after PYCP supplementation. Collectively, PYCP can activate downstream targets of the Akt/mTORC1 signaling pathway by activating IGF-I, supporting the role of this signaling pathway in the muscle atrophy prevention mechanism of PYCP.

Recent studies have shown that FoxO transcription factors are essential in regulating skeletal muscle atrophy both in vitro and in vivo [53,54]. In skeletal muscle, FoxO1 and FoxO3a transcription factors upregulate the expression of E3 ubiquitin ligases, atrogin-1/MAFbx, and MuRF1, and induce muscle atrophy by increasing muscle protein degradation [22,27]. The present study demonstrated that FoxO nuclear translocation and E3 ubiquitin ligase (atrogin-1/MAFbx and MuRF1) expression levels were increased by DEX exposure, which were reduced by PYCP treatment. Similarly, 20S proteasomal activity was increased by DEX exposure, which was prevented by PYCP treatment.

In addition, FoxO transcription factors activated by the inhibition of the IGF-I/Akt signaling pathway stimulate the autophagy-lysosome pathway via a transcription-dependent mechanism and increase the transcription of proteins related to autophagy [55]. Studies have demonstrated that DEX exposure increases FoxO nuclear translocation in skeletal muscle and activates autophagy-lysosome pathways [56,57]. In the present study, DEX-induced expression of cathepsin-L and autophagy-related genes were downregulated by PYCP supplementation. These results suggest that PYCP prevents DEX-induced muscle atrophy by inhibiting the ubiquitin-proteasome and autophagy-lysosome pathways via the inhibition of FoxO-related atrogenes in skeletal muscle. Thus, the assembled proteolytic system predicts DEX-induced muscle atrophy, and these findings might suggest proteolytic system inhibition as a potential therapeutic strategy for muscle atrophy.

Taken together, our results showed that PYCP prevented the DEX administration-induced skeletal muscle atrophy by regulating IGF-I/Akt/m TORC1/FoxO signaling pathway and proteolytic systems. Therefore, IGF-I/Akt/mTORC1 signaling pathway and proteolytic systems may be critical to PYCP’s prevention of DEX muscle atrophy. This understanding may provide new information for therapies aiming to prevent skeletal muscle atrophy induced by DEX exposure.

## 4. Materials and Methods 

### 4.1. Experimental Animals

All experiments were performed using 8-week-old male C57BL/6 mice weighing 22–23 g. All animals were housed two or three per cage in a temperature—(22–24 °C) and humidity—(40–45%) controlled room. The light:dark cycle was 12 h/12 h and normal rodent pellet diet and water were supplied during the study ad libitum.

### 4.2. Experimental Scheme 

Experiments were performed following treatment with 150 mg/kg and 300 mg/kg PYCP, which is the optimum concentration without toxicity as described by Choi et al. [33]. Following one week of acclimatization, 20 mice were randomly divided into four groups of five: (i) intact vehicle control, (ii) DEX control, (iii) DEX treatment with 150 mg/mL PYCP, and (iv) DEX treatment with 300 mg/mL PYCP. Muscle atrophy was induced via an intraperitoneal injection of DEX (3 mg/kg body weight, once a day for 18 days according to an established method). Two concentrations of PYCP (150 and 300 mg/kg body weight) were orally administered twice a day for 18 days. Equal volumes of distilled water were orally administered to the mice in the intact vehicle control and DEX control groups, instead of PYCP, and saline was intraperitoneally injected into the mice in the intact vehicle control group instead of DEX. This experiment was conducted according to the international regulations of the usage and welfare of laboratory animals and approved by the University Animal Care and Use Committee guidelines at Pukyong National University (Busan, Korea) (Approval No. 2017-18). One day after the final DEX and PYCP treatment, the mice were anesthetized with ether and sacrificed. Immediately thereafter, blood was collected and the hind limb muscles (i.e., gastrocnemius, soleus, and tibialis anterior muscles) were excised. The blood samples were centrifuged at 1000× *g* for 10 min, and serum was collected and stored at −70 °C until further use. The isolated muscle tissues were frozen immediately in liquid nitrogen and stored at −70 °C until analysis.

### 4.3. Measurement of Body Weight and Calf Thickness

The body weight of all mice was measured 1 day before the experiment and daily at 11:00 a.m. during the treatment period using an automatic electronic balance machine (Radwag, Random, Poland). In addition, the left hind calf thickness of all mice was measured before sacrifice using an electronic digital caliper (Mitutoyo Italiana SRL, Lainate, Italy) and recorded in terms of millimeters per mouse.

### 4.4. Measurement of Gastrocnemius, Soleus, and Tibialis Anterior Muscle Weight

The gastrocnemius, soleus, and tibialis muscles were carefully separated from the tibia and fibula. The weights of individual gastrocnemius, soleus, and tibialis muscles were measured (g absolute wet weight) using an automatic electronic balance machine, and the relative weight (% body weight) was calculated to reduce the differences among individual body weights using the body weight at sacrifice and absolute weight as follows: relative muscle mass weight (% body weight) = [(absolute organ weight/body weight at sacrifice) × 100].

### 4.5. Measurement of Glucose Uptake

Glucose levels in serum were determined using a glucose ELISA kit (Asan Pharmaceutical Co., Ltd., Gyeonggi, Korea) according to the manufacturer’s instructions. Briefly, an enzyme solution was added to the serum and maintained at 37 °C for 5 min in a 5% CO_2_ humidified atmosphere. The absorbance at 500 nm was measured within 40 min.

### 4.6. Measurement of Creatine Kinase Activity

Mouse serum CK production was quantitatively analyzed based on the double-sandwich ELISA technique using a mouse CK ELISA kit (MyBiosource, San Diego, CA, USA) according to the manufacturer’s instructions. In brief, 0.1 mL of each appropriately diluted serum sample was transferred to an empty well of a 96-well plate and incubated at 37 °C for 90 min. Following incubation, 0.1 mL of biotinylated mouse CK antibody liquid was added to the wells and incubated at 37 °C for 60 min, after which unbound conjugates were removed by washing three times with phosphate-buffered saline (PBS), allowing the washing buffer to sit in the wells for 1 min before removing. After washing, 0.1 mL of prepared enzyme-conjugate liquid was transferred to each well and the plate was incubated at 37 °C for 30 min, followed by washing with PBS as described above. Next, 0.1 mL of tetramethylbenzidine (TMB) color developing agent was added to each well and the plate was incubated at 37 °C in the dark for 30 min. The TMB color reaction was catalyzed with horseradish peroxidase to produce a blue-colored product that turned yellow after adding TMB stop solution (1 N H_2_SO_4_). The optical density of yellow was proportional to the mouse serum CK concentration, which was measured by absorbance at 450 nm in a microplate reader within 30 min of adding the stop solution.

### 4.7. Measurement of Lactate Dehydrogenase Activity

Mouse serum LDH production was quantitatively analyzed based on the double-sandwich ELISA technique using a mouse LDH ELISA kit (MyBiosource, San Diego, CA, USA) according to the manufacturer’s instructions. In brief, 0.1 mL of each appropriately diluted serum sample was transferred to an empty well of a 96-well plate and incubated at 37 °C for 90 min. Following incubation, 0.1 mL of biotinylated mouse LDH antibody liquid was added to the wells and incubated at 37 °C for 60 min, after which unbound conjugates were removed by washing three times with phosphate-buffered saline (PBS), allowing the washing buffer to sit in the wells for 1 min before removing. After washing, 0.1 mL of prepared enzyme-conjugate liquid was transferred to each well and the plate was incubated at 37 °C for 30 min, followed by washing with PBS as described above. Next, 0.1 mL of tetramethylbenzidine (TMB) color developing agent was added to each well and the plate was incubated at 37 °C in the dark for 30 min. The TMB color reaction was catalyzed with horseradish peroxidase to produce a blue-colored product that turned yellow after adding TMB stop solution (1 N H_2_SO_4_). The optical density of yellow was proportional to the mouse serum LDH concentration, which was measured by absorbance at 450 nm in a microplate reader within 30 min of adding the stop solution.

### 4.8. Preparation of Total Cell Lysates

Gastrocnemius muscle tissue was minced with a tissue homogenizer in RIPA buffer [1% NP-40, 0.25% sodium deoxycholate, 1 mM ethylene glycol-bis (β-aminoethyl ether)-*N*,*N*,*N*′,*N*′-tetraacetic acid, 150 mM NaCl, and 50 mM Tris-HCl; pH 7.5] containing protease inhibitors (1 mg/mL aprotinin, 1 mg/mL leupeptin, 1 mg/mL pepstatin A, 200 mM Na_3_VO_4_, 500 mM NaF, and 100 mM PMSF) for 1 min and incubated for 30 min on ice. The extracts were centrifuged at 16,000× *g* for 10 min at 4 °C, and the supernatants were collected and protein concentrations were quantified using a BCA protein assay kit (Thermo Fisher Scientific, Inc., Rockford, IL, USA) according to the manufacturer’s instructions. Then, the supernatant was used in the Western blot analysis.

### 4.9. Preparation of Cytosolic and Nuclear Extracts

Gastrocnemius muscle tissues were minced with a tissue homogenizer in hypotonic lysis buffer [25 mM HEPES (pH 7.5), 5 mM EDTA, 5 mM MgCl_2_, and 5 mM DTT] containing protease inhibitors (1 mg/mL aprotinin, 1 mg/mL leupeptin, 1 mg/mL pepstatin A, 200 mM Na_3_VO_4_, 500 mM NaF, and 100 mM PMSF) for 1 min and incubated for 15 min on ice. NP-40 (2.5%) was added and the cells were lysed for an additional 10 min. Nuclei were collected by centrifugation at 7500× *g* for 15 min at 4 °C. The supernatant was collected as the cytosolic fraction. Nuclear proteins were resuspended in extraction buffer [10 mM HEPES (pH 7.9), 100 mM NaCl, 1.5 mM MgCl_2_, 0.1 mM EDTA, and 0.2 mM DTT] and incubated for 20 min at 4 °C. The extracts were centrifuged at 16,000× *g* for 10 min, and the protein levels were determined using a BCA protein assay kit (Thermo Fisher Scientific, Inc., Rockford, IL, USA) according to the manufacturer’s instructions. The supernatant was used for the Western blot analysis.

### 4.10. Western Blot Analysis

Equal amounts of proteins (30 μg) were separated with 6–12.5% sodium dodecyl sulfate-polyacrylamide gel electrophoresis and transferred onto a polyvinylidene fluoride membrane (Millipore, Bedford, MA, USA). The membrane was blocked at room temperature with 1% bovine serum albumin in TBS-T (10 mM Tris-HCl, 150 mM NaCl, and 0.1% Tween-20) and incubated with the prescribed primary antibodies (Table 2). The secondary antibodies (diluted 1:10,000 to 1:20,000) included horseradish peroxidase-conjugated anti-rabbit IgG (7074S; Cell Signaling Technology, Inc., Beverly, MA, USA), donkey anti-goat IgG (A50-101P; Bethyl Laboratories, Inc., Montgomery, TX, USA), and goat anti-mouse IgG (sc-2031; Santa Cruz Biotechnology, Inc., Santa Cruz, CA, USA) antibodies. Signals were detected using an enhanced chemiluminescence Western blot analysis kit (Thermo Fisher Scientific, Inc., Rockford, IL, USA). The experiments were performed in triplicate and densitometry analysis was performed using Multi-Gauge software ver. 3.0 (Fujifilm Life Science, Tokyo, Japan).

### 4.11. Real-Time Polymerase Chain Reaction

The mRNA expression levels of specific genes were evaluated with real-time PCR. Total RNA was isolated from gastrocnemius muscle tissue using TRIzol reagent (Invitrogen Life Technologies, Carlsbad, CA, USA). The resulting RNA was evaluated by measuring the absorbance at 260 and 280 nm to determine the RNA concentration and purity, respectively. A RevoScript Reverse Transcriptase PreMix Kit (Intron Biotechnology Co. Ltd., Seongnam, Korea) was used to prepare cDNA according to the manufacturer’s instructions, and the samples were stored at −50°C. Real-time PCR was conducted in 20-μL reactions using a QuantiMix SYBR kit (PhilKorea Technology, Inc., Daejeon, Korea) and an Illumina Eco real-time PCR system (Illumina, Inc., Hayward, CA, USA). All mRNA levels were normalized using GAPDH as an internal control. The primers used for the amplification are listed in Table 3.

### 4.12. Measurement of 20S Proteasome Activity

The chymotrypsin-like activity of the 20S proteasome was measured based on changes in the fluorescence of AMC conjugated to the chymotrypsin peptide substrate LLVY using a 20S proteasome activity assay kit (Chemicon, Temecula, CA, USA). In brief, gastrocnemius muscle tissues were suspended in RIPA lysis buffer [50 mM Tris-HCl (pH 7.5), 150 mM NaCl, 0.5% sodium deoxycholate, 1% Triton X-100, 0.1% sodium dodecyl sulfate, and 2 mM EDTA] containing protease inhibitors (1 mg/mL aprotinin, 1 mg/mL leupeptin, 1 mg/mL pepstatin A, 200 mM NA_3_VO_4_, 500 mM NaF, and 100 mM PMSF) and centrifuged at 16,000× *g* for 10 min at 4 °C. The protein concentration in the supernatant was determined using a BCA protein assay kit (Thermo Fisher Scientific, Inc., Rockford, IL, USA). The supernatant was incubated for 90 min at 37 °C with a labeled substrate, LLVY-7-amino-methylcoumarin, and the cleavage activity was monitored by detection of the free fluorophore AMC using a fluorescence plate reader (Gen5 ELISA, Bio-Tek, Houston, TX, USA).

### 4.13. Statistical Analysis

The mean values were compared with analysis of variance using SPSS ver. 10.0 software (SPSS Inc., Chicago, IL, USA). The values are presented as the means ± standard deviation. Groups denoted with different letters differed significantly according to Duncan’s multiple-range test.

## Figures and Tables

**Table 1:** Effect of PYCP supplementation on body weight (BW) changes in C57BL/6 mice.

	Group			
	CON	DEX	DEX + PYCP150	DEX + PYCP300
Basal BW (g)	24.13 ± 0.69	24.04 ± 0.41	24.69 ± 0.97	24.17 ± 0.84
Final BW (g)	25.77 ± 0.41	24.35 ± 0.57 *	25.22 ± 1.01 ^#^	25.47 ± 0.65 ^#^

The results are presented as the means ± standard deviation of five mice. * *p* < 0.05 vs. the corresponding control group; ^#^ *p* < 0.05 vs. the corresponding DEX-only treatment group.

**Table 2:** Primary antibodies (monoclonal or polyclonal) used in the western blot analysis.

Antibody	Manufacturer and Catalog No.	Species of Origin	Dilution Rate
4E-BP1	Santa Cruz Biotechnology: sc-9977	Mouse	1:1000
Akt	Santa Cruz Biotechnology: sc-8312	Rabbit	1:1000
ATG5	Santa Cruz Biotechnology: sc-33210	Rabbit	1:1000
ATG7	Santa Cruz Biotechnology: sc-376212	Mouse	1:1000
ATG12	Santa Cruz Biotechnology: sc-271688	Mouse	1:1000
Atrogin-1/MAFbx	Santa Cruz Biotechnology: sc-27645	Goat	1:2000
β-actin	Santa Cruz Biotechnology: sc-47778	Mouse	1:1000
BECN1	Santa Cruz Biotechnology: sc-11427	Rabbit	1:1000
Cathepsin-L	Santa Cruz Biotechnology: sc-6498	Goat	1:1000
eIF4E	Santa Cruz Biotechnology: sc-514875	Mouse	1:1000
FoxO1	Santa Cruz Biotechnology: sc-374427	Mouse	1:500
FoxO3a	Santa Cruz Biotechnology: sc-9813	Goat	1:1000
GAPDH	Santa Cruz Biotechnology: sc-25778	Rabbit	1:1000
IGF-IR	Santa Cruz Biotechnology: sc-713	Rabbit	1:1000
IRS-1	Santa Cruz Biotechnology: sc-560	Rabbit	1:1000
Lamin B	Santa Cruz Biotechnology: sc-377000	Mouse	1:1000
LC3-I/II	Cell Signaling: #4108S	Rabbit	1:1000
mTOR	Santa Cruz Biotechnology: sc-8319	Rabbit	1:1000
MuRF1	Santa Cruz Biotechnology: sc-27642	Goat	1:2000
p62/SQSTM1	Cell Signaling: #5114S	Rabbit	1:1000
p-4E-BP1	Santa Cruz Biotechnology: sc-293124	Mouse	1:1000
p70S6K	Santa Cruz Biotechnology: sc-8418	Mouse	1:1000
p-Akt	Santa Cruz Biotechnology: sc-135650	Mouse	1:500
p-FoxO1	Cell Signaling: #9461S	Rabbit	1:500
p-FoxO3a	Cell Signaling: #9466S	Rabbit	1:1000
p-IGF-IR	Santa Cruz Biotechnology: sc-101703	Rabbit	1:1000
p-IRS-1	Santa Cruz Biotechnology: sc-17200	Goat	1:1000
p-mTOR	Santa Cruz Biotechnology: sc-293132	Mouse	1:1000
p-p70S6K	Santa Cruz Biotechnology: sc-8416	Mouse	1:1000
p-S6	Santa Cruz Biotechnology: sc-293144	Mouse	1:1000
Raptor	Santa Cruz Biotechnology: sc-81537	Mouse	1:1000
Rheb	Santa Cruz Biotechnology: sc-271509	Mouse	1:1000
Rictor	Santa Cruz Biotechnology: sc-81538	Mouse	1:1000
S6	Santa Cruz Biotechnology: sc-74459	Mouse	1:1000

**Table 3:** Oligonucleotide primer sequences used for real-time PCR.

Gene	Accession No.	Sequence (5′–3′)	Amplicon (bp)
Atrogin-1/MAFbx	NM_026346.3	F: ATGCACACTGGTGCAGAGAG R: TGTAAGCACACAGGCAGGTC	168
Cathepsin-L	M20495.1	F: GACCGGGACAACCACTGTG R: CCCATCAATTCACGACAGGAT	61
MuRF1	DQ229108.1	F: TGTCTGGAGGTCGTTTCCG R: GTGCCGGTCCATGATCACTT	59
GAPDH	NM_008084.3	F: ACTCCACTCACGGCAAATTCA R: CGCTCCTGGAAGATGGTGAT	91
